# An Energy-Efficient and Compact Clustering Scheme with Temporary Support Nodes for Cognitive Radio Sensor Networks

**DOI:** 10.3390/s140814634

**Published:** 2014-08-11

**Authors:** Shelly Salim, Sangman Moh, Dongmin Choi, Ilyong Chung

**Affiliations:** Department of Computer Engineering, Chosun University, 309 Pilmun-daero, Dong-gu, Gwangju 501-759, Korea; E-Mails: shellysalim22@gmail.com (S.S.); jdmcc@chosun.ac.kr (D.C.); iyc@chosun.ac.kr (I.C.)

**Keywords:** cognitive radio sensor network, clustering, energy consumption, lifetime, clustering overhead

## Abstract

A cognitive radio sensor network (CRSN) is a wireless sensor network whose sensor nodes are equipped with cognitive radio capability. Clustering is one of the most challenging issues in CRSNs, as all sensor nodes, including the cluster head, have to use the same frequency band in order to form a cluster. However, due to the nature of heterogeneous channels in cognitive radio, it is difficult for sensor nodes to find a cluster head. This paper proposes a novel energy-efficient and compact clustering scheme named clustering with temporary support nodes (CENTRE). CENTRE efficiently achieves a compact cluster formation by adopting two-phase cluster formation with fixed duration. By introducing a novel concept of temporary support nodes to improve the cluster formation, the proposed scheme enables sensor nodes in a network to find a cluster head efficiently. The performance study shows that not only is the clustering process efficient and compact but it also results in remarkable energy savings that prolong the overall network lifetime. In addition, the proposed scheme decreases both the clustering overhead and the average distance between cluster heads and their members.

## 1. Introduction

A wireless sensor network (WSN) is a network of a large number of densely deployed sensor nodes [1]. The sensor nodes in a WSN can monitor a variety of ambient conditions, such as temperature, humidity, and movement. Sensed data are sent to a sink node or a base station. WSNs have various applications in the military, environmental, health, home, and commercial fields. The sensor nodes in WSNs are generally densely deployed in a random placement over remote areas. The sensor nodes need to have self-organizing ability from the topology formation stage until the data collection stage. Clustering is a well-known strategy in WSNs, where nearby nodes form a group called a cluster, and divide their data transmission activities into intra-cluster and inter-cluster transmissions [2]. The clustering technique helps to minimize routing activities, conserve bandwidth, stabilize the network topology, and preserve energy [3]. The sensor nodes are usually battery-powered, which makes their energy resources very limited. Moreover, their data-processing resources are also limited. Unless the sensor nodes have energy harvesting capabilities [4], given the battery capacity limitations, the energy consumption of the sensor nodes determines the lifetime of a WSN.

Recently, research activities in the field of cognitive radio have been increasing rapidly. Usually, a cognitive radio network is assumed to be implemented as a secondary network that exploits the intermittent vacant channels of a primary network [5]. The idea to combine WSN with cognitive radio by integrating cognitive radio at the sensor nodes is a promising one. The resulting network is called a cognitive radio sensor network (CRSN). The CRSN benefits from the advances made in key research areas of cognitive radio such as opportunistic spectrum access and transmission parameter reconfigurability [6]. However, in a CRSN the resource-constrained sensor nodes need to perform the cognitive radio tasks in addition to their original tasks, resulting in increased energy consumption.

**Figure 1.**
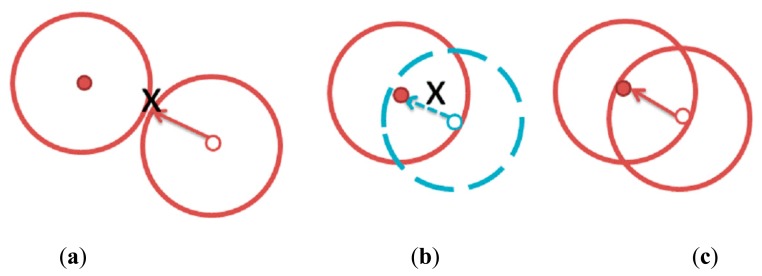
Clustering requirements in a CRSN. The small solid circle represents a cluster head and the small empty circle represents a sensor node attempting to locate a cluster head. The large circles represent the sensor's transmission range. (**a**) The sensor nodes cannot identify the cluster head because the cluster head is located outside its transmission range. (**b**) The sensor node and the cluster head are using different channels and, thus, even though the cluster head is located inside the sensor node's transmission range, the sensor node cannot find the cluster head. (**c**) The sensor node and the cluster head are using the same channel, and the cluster head is located inside the sensor node's transmission range. Thus, the sensor node is able to find the cluster head and join the cluster.

Clustering in a CRSN is similar to clustering in an ordinary WSN. Each cluster consists of one cluster head and a number of cluster members. The cluster members sense the attributes of the target environment and send the sensed data to the cluster head. The cluster head aggregates the data sent from its members and forwards the aggregated data to the data collection entity called the sink or base station. Indeed, clustering is considered a proper topology handling method for a CRSN, primarily because only the cluster heads need to perform cognitive radio management tasks instead of all the sensor nodes, which reduces the total energy consumption. However, clustering in a CRSN has an additional requirement: that is, to form a cluster, the sensor nodes not only have to be in the transmission range of one another but also have to operate in the same communication channel, as illustrated in Figure 1. This limitation might cause a poor cluster formation.

In some works in the field of CRSNs, the clustering methods are assumed or fixed [7–9]. The clustering method is an important aspect as it directly affects the energy consumption and the network lifetime. Several works have reported different clustering methods for CRSNs. Event-driven spectrum-aware clustering [10] creates temporal clusters for each event based on the position, node degree, available channels, and distance to the sink. The clusters are no longer available at the end of the event. Thus, event-driven spectrum-aware clustering is only suitable for WSNs intended for event-driven applications. Distributed spectrum-aware clustering (DSAC) [11,12] uses the local minimum distance obtained by information exchanges to merge two nearby nodes or clusters that share the same available channels. The cluster formation process is repeated until the optimal number of clusters is reached. The adaptation of the low-energy adaptive clustering hierarchy (LEACH) protocol to suit CRSNs was reported in [13].

Fundamentally, clustering in CRSNs should consider the energy consumption of the sensor nodes because this directly affects the network lifetime. In the same approach, clustering overhead has to be minimized in order to achieve efficient power consumption, while supporting both event-driven and regular data collection. Motivated by providing a suitable clustering method for CRSNs, as described above, we propose a novel energy-efficient and compact clustering scheme called clustering with temporary support nodes (CENTRE). CENTRE aims to improve the network performances with the deployment of temporary support nodes. Here, we present the main features of CENTRE:

- Temporary support node: The cluster head assigns one sensor node called the temporary support (TS) node to serve as a support node for a short time. The TS node broadcasts an invitation packet on each channel. Because the cluster head should stay on its operating channel to accept sensor nodes' registration, it needs the help of the temporary support node to send out the invitation. The invitation packet contains the information about the existence and operating channel of the cluster head of the TS node. As each sensor node might tune to a different channel, the sensor node might be unable to discover a cluster head even though the cluster head is located within the sensor node's transmission range. Therefore, the role of the TS node is to alert the sensor nodes that have not joined any cluster about the presence of the TS node's cluster head.
- Two sub-phases of cluster formation: The cluster formation process consists of two sub-phases: cluster head discovery and cluster member invitation. In CRSNs, it is difficult for sensor nodes to find a cluster head. The two sub-phases enable the sensor nodes to find a cluster head effectively.
- Partial spectrum sensing: The sensor nodes do not carry out the spectrum sensing process on all the channels but only on some part of the channels to conserve energy and time. This is not a usual approach because the sensor nodes are usually required to perform full spectrum sensing or even cooperative spectrum sensing [14,15]. In our proposed work, the sensor nodes intentionally perform partial spectrum sensing to save energy because CRSN is an energy-constrained network.
- Communication frequency selection: Intra-cluster transmissions are assigned a high frequency, whereas the inter-cluster transmissions are assigned a low frequency. High frequency channels support higher data transmission rates but shorter transmission ranges and, thus, they are suitable for data transmission between a cluster head and the cluster members. Low frequency channels, on the other hand, support longer transmission ranges and consume less energy and, thus, they are suitable for one-hop data collections from cluster heads to the sink.

In CENTRE, the following assumptions are made:

- The deployment of sensor nodes is random, dense, and redundant. This assumption implies that a number of sensor nodes might be excluded (put to sleep) during the data collection activity, without affecting the WSN's sensing coverage functionality.
- Each sensor node is equipped with one cognitive radio transmitter.
- When a sensor node (of any class) simultaneously receives more than one packet, it receives one packet successfully while discarding the others.
- The medium access control protocol is based on time division multiple access (TDMA).
- The CRSN application requires periodic data collection and there is at least one reserved, low-frequency common control channel between the sink and the cluster heads. However, there is no common control channel among the high-frequency channels.
- The CRSN is deployed in a remote location where no primary user is present. Even though there is no primary user, the network could be considered a CRSN because the sensor nodes are equipped with cognitive radio capabilities such as dynamic spectrum access and transmission parameters reconfigurability. We are aware that wireless networks employing cognitive radios should consider the interference to the primary users. Indeed, we plan to include primary users in the future work (Section 4), but in this paper, our main objective is to introduce our novel approach for clustering that involves a particular cluster member to be a temporary support node and to evaluate its performance. Even though we do not include primary users, our underlying network still can be categorized as a CRSN because it is a wireless sensor network and the sensor nodes are equipped with cognitive radio capability, according to the definition of cognitive radio given in [5].

The principal contribution of this paper is the development of a clustering method suitable for CRSNs with low energy consumption. Besides energy conservation, the proposed work is shown to have low clustering overhead and a short distance between cluster heads and their members. Another contribution is that a novel approach of clustering is developed by introducing the concept of a temporary support node. With the help of this temporary support node, the proposed work is able to perform well under CRSN environment. The rest of this paper is organized as follows: Section 2 explains the proposed CENTRE algorithm and its features. In Section 3, the performance of CENTRE is evaluated. Finally, Section 4 presents the conclusions drawn.

## 2. Clustering with Temporary Support Nodes (CENTRE)

CENTRE, the clustering method designed for CRSNs, is performed in rounds. Each round consists of two phases: the cluster formation phase and the data transmission phase. The cluster formation phase consists of two sub-phases: cluster head discovery (henceforth called sub-phase 1) and cluster member invitation (henceforth called sub-phase 2). The durations of each phase and sub-phase are fixed and predetermined.

**Figure 2.**
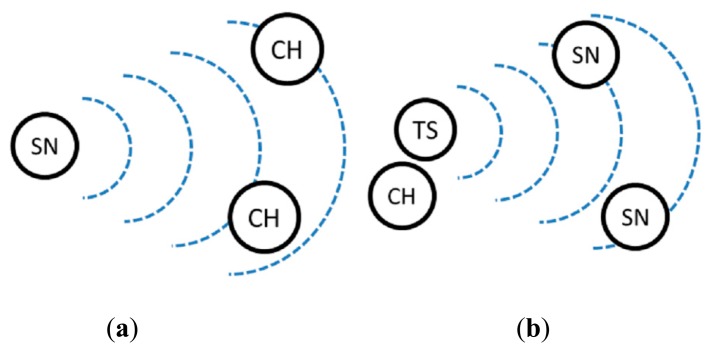
Cluster formation phase. (**a**) Sub-phase 1: cluster head discovery, when a sensor node tries to locate cluster heads. (**b**) Sub-phase 2: cluster member invitation, when a cluster head tries to invite new cluster members with the help of a temporary support node. (SN: sensor node, CH: cluster head, TS: temporary support).

**Figure 3.**
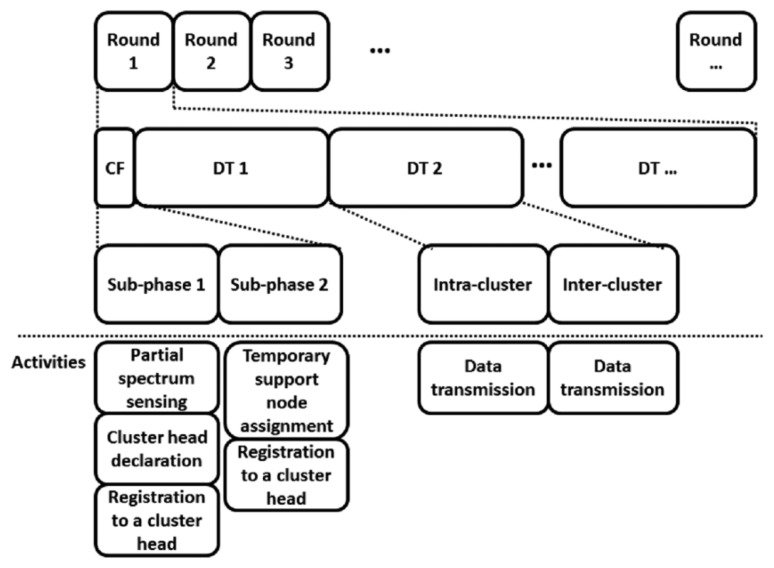
The procedures in CENTRE rounds and the activities performed during the rounds. (CF: cluster formation, DT: data transmission).

In sub-phase 1, the sensor nodes search for the cluster head. In a CRSN, however, the sensor nodes might not be able to locate a cluster head even though the cluster head may be inside their transmission range because the sensor nodes and the cluster head use different channels. This condition is anticipated in sub-phase 2 when each cluster head actively search for sensor nodes that can become its members, with the help of a TS node. Figure 2 illustrates the aim of cluster formation sub-phases 1 and 2. The procedures of the CENTRE rounds will be shown in Figure 3 later.

After the completion of the cluster formation phase, the clustering process is completed with one cluster head in each cluster. The data transmission phase that follows involves multiple pairs of intra-cluster and inter-cluster data transmission. During the intra-cluster data collection, the cluster members send the sensed data (related not to spectrum sensing, but to application-related environment sensing) to their cluster heads using one of the high-frequency channels agreed upon with the cluster head. During the inter-cluster data transmission, the cluster heads send the data to the sink by one-hop transmission using one of the low-frequency channels assigned by the sink.

The sensor nodes are divided into four classes:

1. Sensor node that does not belong to any cluster, simply sensor node (SN)
2. Cluster head (CH)
3. Sensor node that connects to a cluster head, namely cluster member (CM)
4. Temporary support (TS) node

During the initial deployment, all sensor nodes are in the class SN. By adopting CENTRE, some sensor nodes would become CHs and CMs by the end of cluster formation sub-phase 1 while the rest stay as SNs. During cluster formation sub-phase 2, a number of CMs might be selected to act as TSs (maximum one TS per cluster). When the tasks of the TSs are completed, they return to function as CMs. However, at the end of sub-phase 2, some sensor nodes might still be SNs; in other words, they do not belong to any cluster. These SNs will be unable to participate in the following data transmissions. However, by assuming a dense and redundant deployment of the sensor nodes in the network, the sensing coverage is expected to be tolerable.

The five major activities in CENTRE are given below.

1. *Partial spectrum sensing and cluster head discovery*: The sensor nodes carry out the spectrum sensing process on some of the channels to find cluster heads.
2. *Cluster head declaration*: The sensor nodes declare themselves as cluster heads after they fail to discover any cluster head on their current operating channels by following a predetermined probability.
3. *Registration to a cluster head*: The sensor nodes that find a cluster head proceed to join the cluster.
4. *TS node assignment and cluster member invitation*: The cluster heads might assign the closest cluster member as a support node temporarily. The TS node broadcasts invitation packets on each channel.
5. *Data transmission*: Data transmission includes both intra-cluster and inter-cluster data transmissions.

These activities are performed regularly during the CENTRE rounds, as illustrated in Figure 3.

### 2.1. Cluster Formation Phase

**Figure 4.**
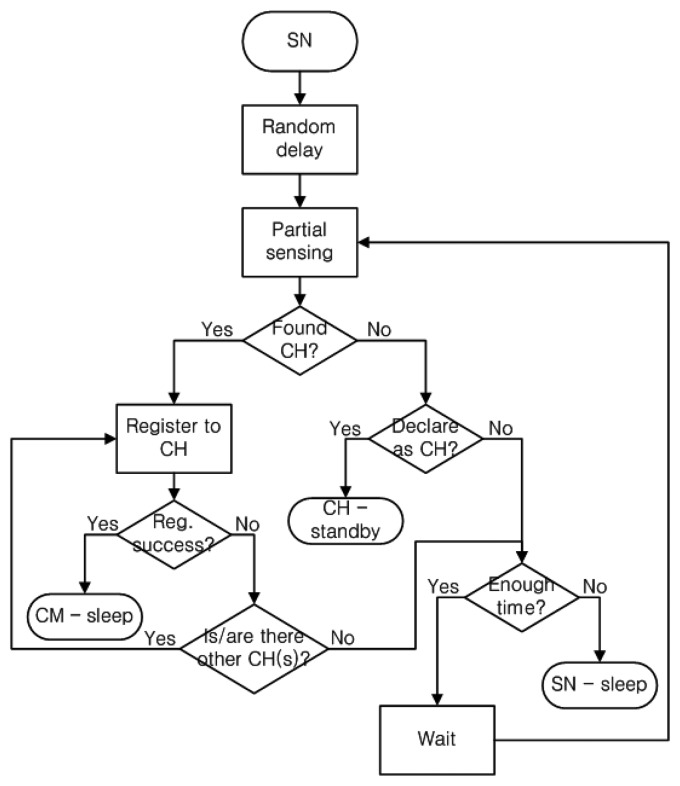
Procedural flow of the cluster formation phase.

The cluster formation phase aims to build an optimal cluster topology in a distributive manner. As mentioned earlier, this phase consists of two sub-phases: cluster head discovery (sub-phase 1) and cluster member invitation (sub-phase 2). In sub-phase 1, the sensor nodes search for cluster heads, whereas, in sub-phase 2, the cluster heads search for new cluster members, with the help of TS nodes. Figure 4 shows the flow chart of the cluster formation phase.

Three activities take place in sub-phase 1. They are: (1) partial spectrum sensing and cluster head discovery, (2) registration of sensor nodes to a cluster head, and (3) cluster head declaration. The first step in cluster formation is partial spectrum sensing, in which the sensor nodes perform spectrum sensing on a part of the entire spectrum. The purpose of partial spectrum sensing is to save energy and time, because sensing on the entire spectrum requires considerably higher processing tasks and time, but the sensor nodes have only limited resources. The sensor nodes start partial spectrum sensing after a random delay. The random delay is applied to increase the probability of discovering a cluster head. If all the sensor nodes start spectrum sensing at the same initiation time, then no cluster head would be found because all the sensor nodes are performing spectrum sensing. If a sensor node waits, then by the time it starts spectrum sensing, there is a possibility of it finding a cluster head as some sensor nodes might have declared themselves as cluster heads earlier. However, the random delay is kept short to minimize the cluster formation duration.

**Figure 5.**
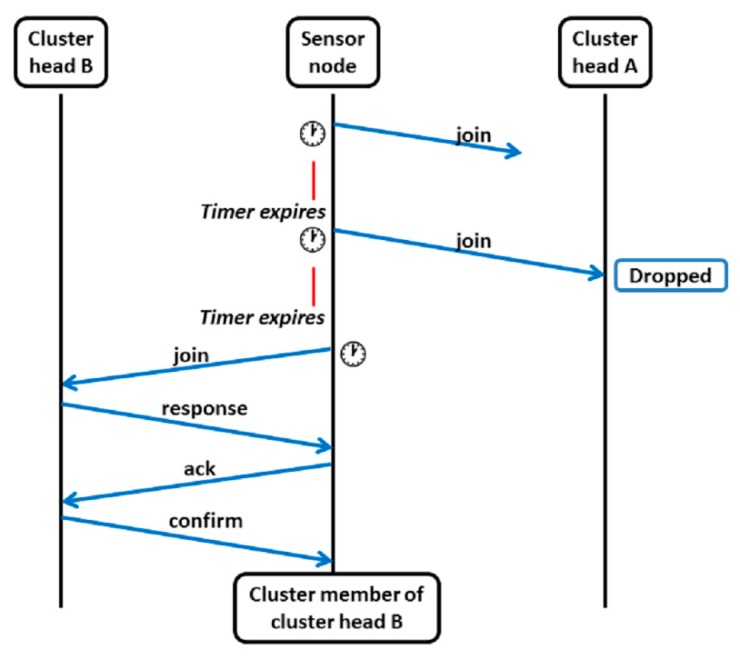
Procedure for registration to a cluster head.

Each sensor node keeps a list of cluster heads that it has found during partial spectrum sensing and, the sensor node registers to the first-listed cluster head. No additional computation is performed to select among cluster heads from the list to reduce energy consumption. The packet exchanges that take place between a sensor node and the cluster heads during the registration period is shown in Figure 5.

The sensor node sends a join packet to the cluster head first on the list and sets its timer. The join packet may fail to reach the cluster head or collide with other packets at the cluster head. Hence, if the timer expires but the sensor node has not received a response packet, it sends a join packet to the same cluster head one more time. When the second join packet also fails to elicit a response from the first cluster head, the sensor node sends a join packet to the next cluster head on the list. The cluster head that receives the join packet sends back a response packet to the sensor node. Here, it is assumed that when a cluster head receives many join packets simultaneously, one join packet is received successfully and the other join packets are dropped. Moreover, the cluster head does not send any notification to the sending nodes about the dropped join packets.

Once the sensor node receives a response packet from the cluster head, it calculates the distance and required transmission power based on the received power level. The sensor node reconfigures its transmission power level to the minimum required power level (reconfiguration is enabled by cognitive radio) and sends an acknowledgement packet to the cluster head at the new transmission power level. The acknowledgement packet also contains the distance information. The purpose of the acknowledgement packet is to ensure that the cluster head can successfully receive and decode a packet transmitted at the new transmission power level. The distance information is used during sub-phase 2. When the cluster head receives the acknowledgement packet, it replies with a confirmation packet, sets the sensor node as its cluster member, and records its distance information. Similarly, the sensor node sets itself as the cluster member of the respective cluster head after it receives the confirmation packet. Then, the cluster member goes to the sleep state until the end of sub-phase 1.

In case the registration fails and there is no cluster head on the list, the sensor node checks the remaining time (the duration of sub-phase 1 is fixed and predetermined). If the remaining time is sufficient to support another round of partial spectrum sensing and registration trial, then the sensor node waits for a random delay period and repeats partial spectrum sensing. If the remaining time is insufficient, then the sensor node goes to the sleep state until the end of sub-phase 1.

If no cluster head is found at the end of partial spectrum sensing, then the sensor node declares itself as a cluster head with a certain predetermined probability. When the sensor node becomes a cluster head, it beacons about its presence periodically on its operating channel and stays ready to process registration requests. When the sensor node fails to become a cluster head, it again checks the remaining time. If the time is sufficient, it waits and repeats partial spectrum sensing; otherwise, it goes to the sleep state until the end of sub-phase 1.

Sub-phase 2 starts with the assignment of TS nodes. Each cluster head computes the number of cluster members that belong to the cluster. If the cluster head does not have any member, then it becomes a sensor node. If the number of members in the cluster is less than the predefined threshold, then the cluster head assigns the closest cluster member as a TS node; otherwise, the cluster head and its members go to the sleep state until the end of sub-phase 2. As the cluster head has recorded the distance information between itself and each of its cluster members during the registration procedure, it easily decides the closest cluster member. The cluster head then sends a TS node assignment packet to that cluster member and stays ready to process registration requests from sensor nodes until the end of sub-phase 2.

The consideration to assign the closest cluster member as the temporary support node is as follows: as the closest cluster member/temporary support node broadcasts an invitation, the sensor nodes within its transmission range could receive it. However, these sensor nodes need to transmit their registration packet to the cluster head, not to the temporary support node. Because the temporary support node is the closest node to the cluster head, if a sensor node can receive a packet form a temporary support node, then it is highly probable that it can send a packet successfully to the cluster head.

Each cluster member wakes up from the sleep state and waits for the TS node assignment packet from the cluster head. If the cluster member receives a TS node packet, then it becomes a TS node. Otherwise, the cluster member recalls its distance to the cluster head. The cluster members that are relatively closer to the cluster head go to the sleep state until the end of sub-phase 2, whereas the cluster members that are farther from the cluster head stay in the active state.

**Figure 6.**
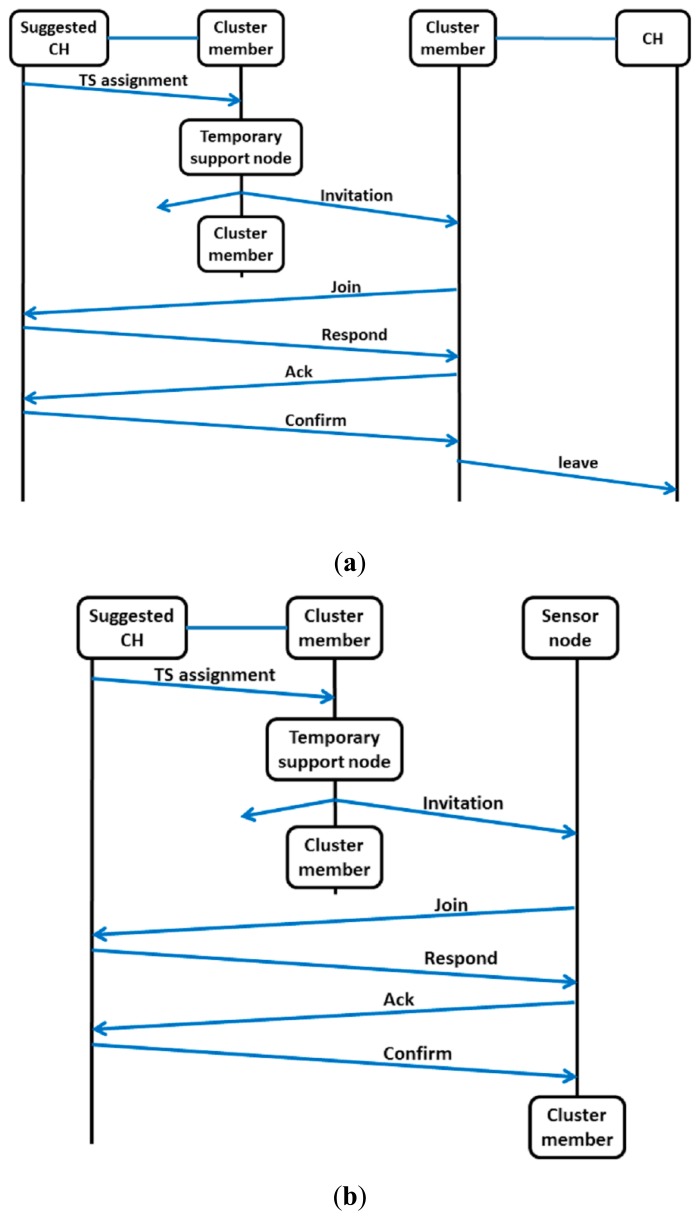
Packet exchanges during sub-phase 2 when the receivers of the invitation packets are (**a**) active cluster members and (**b**) sensor nodes.

The TS node sets its transmission power level to the default setting (maximum) and broadcasts an invitation packet on each available channel. The invitation packet contains the address and operating channel of the TS node's cluster head. After the TS node finishes the broadcasts, it returns to its original class (*i.e.*, becomes a cluster member) and goes to sleep until the end of sub-phase 2. The receivers of the invitation packets are active cluster members and sensor nodes. The cluster head of the TS node is called the suggested cluster head. The receivers of the invitation packets record up to two invitation packets. The exchange of packets during sub-phase 2 is shown in Figure 6.

In case the receiver is a cluster member, it computes the distance to the TS node based on the power level of the received invitation packet. The cluster member considers this distance as the distance to the suggested cluster head because the TS node is the closest node to the suggested cluster head. The cluster member compares the distance to the suggested cluster head with the distance to the current cluster head. If the suggested cluster head is closer than the current cluster head, then the cluster member tries to register to the suggested cluster head by following the registration procedures. When the registration to the suggested cluster head is approved, the cluster member joins the cluster of the suggested cluster head and sends a leave packet to the previous cluster head to inform that it has left that cluster. Otherwise, the cluster member stays with the current cluster head and goes to the sleep state until the end of sub-phase 2.

The sensor node that receives an invitation packet immediately tries to register to the suggested cluster head. If the registration is approved, then the sensor node becomes a cluster member and goes to the sleep state until the end of sub-phase 2. If the registration is unsuccessful, then the sensor node goes to sleep until the end of the current round.

### 2.2. Data Transmission Phase

The data transmission phase consists of pairs of intra-cluster and inter-cluster data transmission repeated multiple times. In intra-cluster data transmission, each cluster member sends its sensed data to the cluster head using a high-frequency channel, whereas in inter-cluster data transmission, each cluster head sends the aggregated data to the sink using a low-frequency channel. By transmitting in low-frequency channel, the cluster heads are able to transmit data to the sink in one-hop transmission.

**Figure 7.**
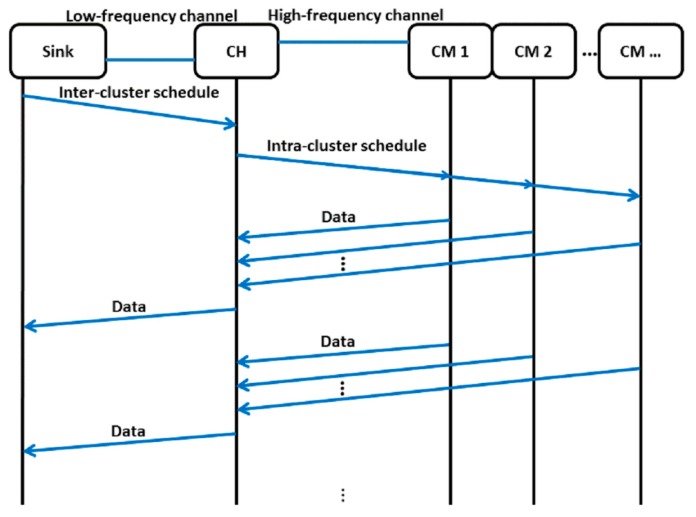
Packet exchanges during the data transmission phase illustrated with one cluster head only.

At the beginning of the data transmission phase, the sink monitors the number of available channels on the low-frequency channels and decides which channels are to be used. The sink creates an inter-cluster schedule, includes the channel information on the schedule packet, and broadcasts the schedule on the low-frequency common control channel. The cluster heads synchronize with each other by receiving the inter-cluster schedule from the sink. After synchronization, the cluster heads switch back to their operating channel, configure the intra-cluster schedule, and broadcast this schedule to their cluster members. The intra-cluster schedule defines the time when a cluster member should report its sensed data to the cluster head. The cluster heads collect the sensed data from the cluster members, aggregate them, and send them to the sink by following its schedule. The inter-cluster and intra-cluster schedules are not updated and are kept unchanged until the end of the round. The packet exchanges during the data transmission phase are shown in Figure 7.

## 3. Performance Evaluation

The CENTRE scheme is evaluated by computer simulation using MATLAB and in comparison with the conventional DSAC scheme [11]. DSAC is selected for comparison because it focuses on clustering in general-purpose CRSNs as in CENTRE. CENTRE is implemented based on time slots, where one time slot equals 18 ms. Hence, DSAC is adjusted to enable simulation based on time slots so that its performance can be compared with that of CENTRE. Moreover, CENRE is implemented based on rounds, where one round consist of cluster formation and data transmission. For CENTRE, a round equals to one time cluster formation and 1000 times intra-cluster and inter-cluster data transmissions pairs or 42,100 time slots. Again, DSAC is adjusted to this time framing. The simulation settings are presented in Table 1.

Four network performance parameters are analyzed in this paper. They are the network lifetime, energy consumption per round, normalized clustering overhead, and average distance between cluster heads and their cluster members. In CRSNs, the network lifetime is the utmost importance metric because there is no constant supply of energy. The sensor nodes have to spend their energy (in the batteries) efficiently to prolong the network lifetime. Energy consumption per round is also analyzed to evaluate the energy consumption trend, in which low and stable energy consumption is desired. Because we propose a clustering method in this paper, we need to measure its effectiveness by measuring the normalized clustering overhead and average distance between cluster heads and the cluster members. To achieve superior performance, the clustering method should have not only low clustering overhead to reduce the energy consumption during cluster formation but also compact clustering (short distance between the cluster heads and the cluster members) to reduce the energy consumption in intra-cluster data transmission.

**Figure 8.**
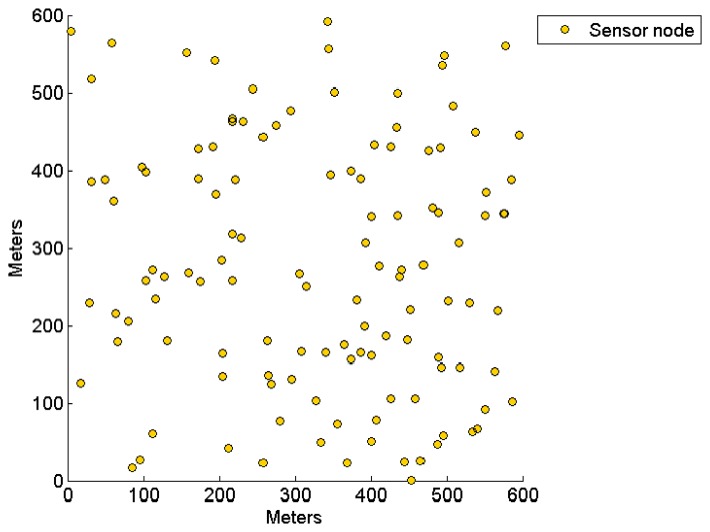
CRSN topology under evaluation. The circles represent the physical location of the sensor nodes in 2D.

The performance parameters are measured at the end of each round. However, as DSAC requires iteration to reach the optimal number of clusters, its cluster formation duration is varied per round. Therefore, the performance parameters of both CENTRE and DSAC are evaluated based on the CENTRE's round duration. The network topology in which sensor nodes are randomly deployed is shown in Figure 8.

**Figure 9.**
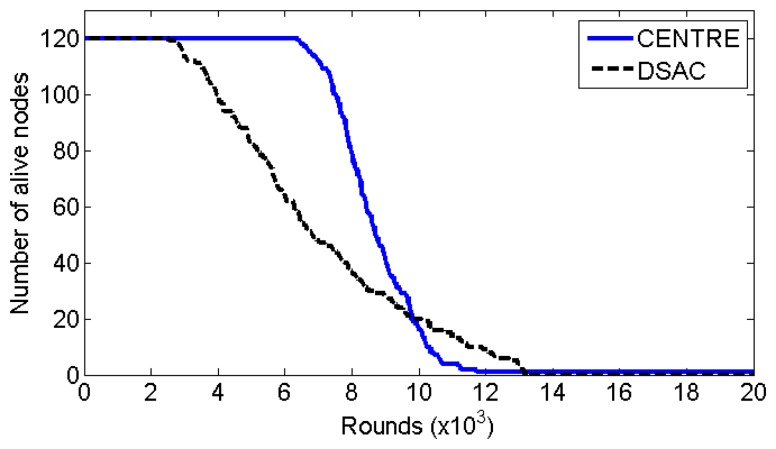
Network lifetime.

Figure 9 shows the number of sensor nodes that are alive per round. Because the sensor nodes are randomly and redundantly deployed, we take the network lifetime definition as the time period during which more than half of the sensor nodes are alive. In other words, the CRSN and its application are considered no longer active when more than half of the sensor nodes have depleted their energy. Because the number of sensor nodes in the simulation is 120, the network lifetime is the time duration from the initial network configuration to the death of the 61st node. As shown in Figure 9, the network lifetime of CENTRE is longer than that of DSAC. The main reasons for this improvement are that CENTRE does not require multiple beacon broadcasts for the nodes/clusters merging iteration during the cluster formation and CENTRE has short, fixed-duration cluster formation. Another reason is that CENTRE enables the adjustment of transmission power to reduce energy consumption. The network lifetime of CENTRE is 34.2% longer than that of DSAC. However, CENTRE has a minor drawback; only a very few number of sensor nodes are alive for a long time. This is because CENTRE is a distributed clustering algorithm without any local information exchange and, thus, the sensor nodes are not aware of the condition of other sensor nodes. Therefore, the sensor nodes would simply follow the algorithm and go to the sleep mode even though the network is no longer active. In real time units, the lifetime of CENTRE is around 74 days (8441 rounds) whereas the lifetime of DSAC is around 55 days (6289 rounds).

**Figure 10.**
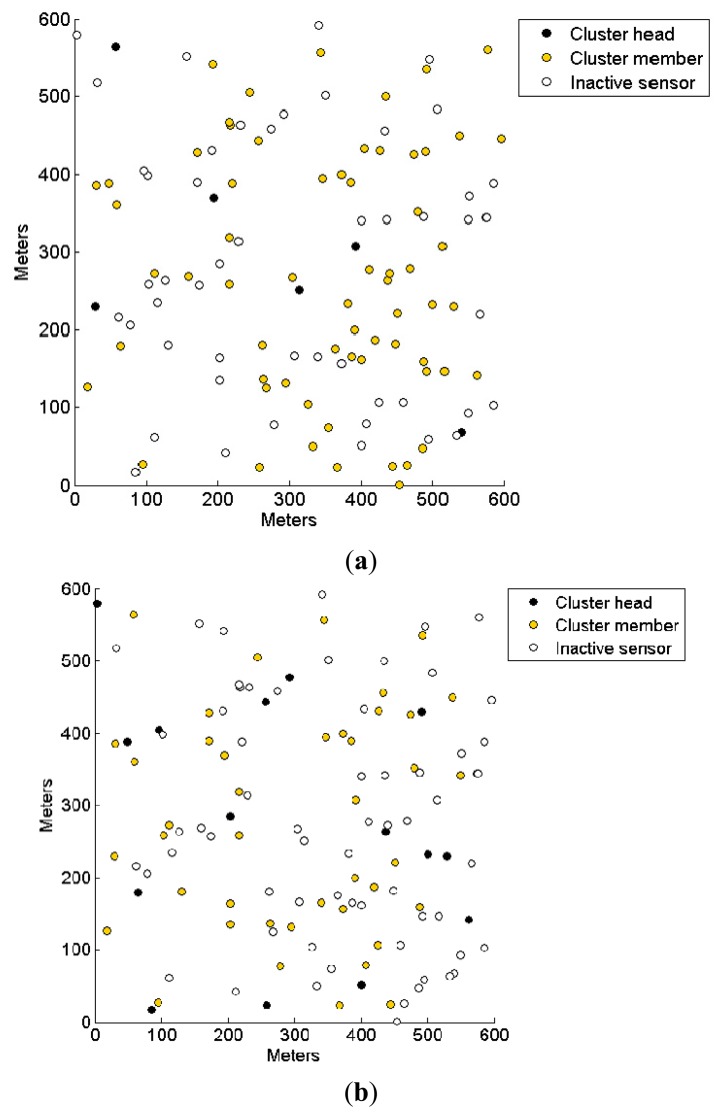
Network composition when half of the sensor nodes are alive for (**a**) CENTRE and (**b**) DSAC.

To further observe the energy consumption and network lifetime, the snapshots of network composition when half of the sensor nodes are alive are shown in Figure 10. The number of cluster heads in DSAC is far higher than that in CENTRE. In DSAC, the number of cluster heads is predetermined and the sensor nodes only have the knowledge of their surroundings (not the global knowledge) by receiving the beacons that their neighboring nodes transmit. In CENTRE, the desired number of clusters is implemented in each sensor node as the probability of becoming a cluster head. Therefore, the number of clusters is approximately the same with the predetermined setting, which is 5% of remaining nodes in our simulation.

**Figure 11.**
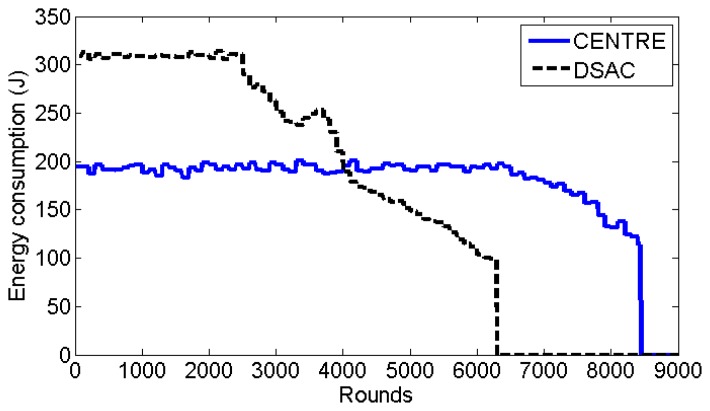
Energy consumption per round (showed until the lifetime of the two schemes).

Figure 11 shows the energy consumption per round for both CENTRE and DSAC schemes. The energy consumption of CENTRE is relatively constant mainly because of the fixed cluster formation duration. Moreover, the organized sleep mode algorithm of CENTRE helps in balancing the energy consumption among the nodes. Until approximately round 2500 (almost half of its lifetime), DSAC consumes 52% more energy than CENTRE. This is mainly due to the fact that, in DSAC, the cluster formation algorithm is based on iterations and multiple beacons are required. When the number of active sensor nodes decreases, the energy consumption of DSAC also decreases. After round 4000, DSAC has lower energy consumption compared to CENTRE. This is because the energy consumption of DSAC is highly dependent on the number of sensor nodes, especially during the cluster formation. On the other hand, the energy consumption of CENTRE is stable throughout the network lifetime. During the network lifetime, on average, the energy consumption per round for CENTRE (185.53 Joule) is 21.1% less than that for DSAC (235.16 Joule).

**Figure 12.**
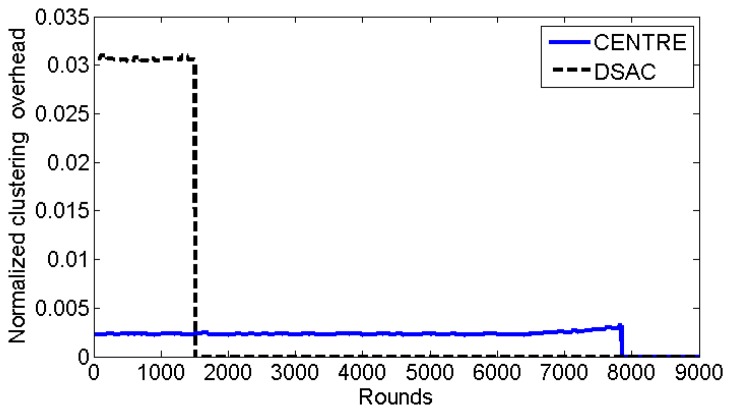
Normalized clustering overhead per round (showed until the lifetime of the two schemes).

To further evaluate the clustering methods, we measure the normalized clustering overhead and the average distance between the cluster heads and their cluster members. The clustering overhead is normalized in terms of time, that is, we define the normalized clustering overhead as the clustering time divided by the data transmission time per round. A small ratio of overhead is desired because it means that the time spent in creating the cluster topology is negligible compared to the actual data transmission time. Figure 12 shows that the normalized clustering overhead of CENTRE is much lower than that of DSAC. On average, the normalized clustering overhead of CENTRE is 0.0024 but that of DSAC is 0.0306 which is 12.8 times higher compared to CENTRE. DSAC has much higher clustering overhead because, again, it is based on iterations requiring higher packets exchanges (beacons). Both schemes have relatively low normalized clustering overheads (less than 0.035). The duration of cluster formation in CENTRE is fixed, which is 100 time slots or equal to 1.8 s. By analyzing the normalized clustering overhead, we could also obtain the required time for clustering in DSAC. Given the normalized clustering overhead and the data transmission time, we could calculate the average duration of cluster formation in DSAC, which is 1250 time slots or 22.5 s according to the definition of the normalized clustering overhead.

**Figure 13.**
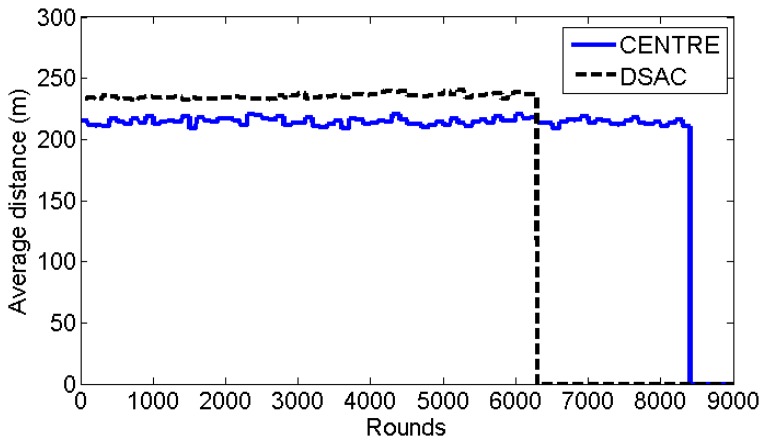
Average distance between the cluster heads and their cluster members (showed until the lifetime of the two schemes).

Figure 13 shows the average distance between the cluster heads and their cluster members. The transmission range of sensor nodes is 300 m. A smaller average distance indicates better cluster formation because it means that each sensor node joins the closest cluster head. Here, CENTRE outperforms DSAC by having about 10% lower average distance. Even though the improvement is only 10%, it is an interesting result because CENTRE performs better than DSAC even though DSAC algorithm merges two closest sensor nodes/clusters into a cluster. This result confirms the efficiency of CENTRE's TS nodes that invite sensor nodes within the transmission range and allow cluster members to switch to another cluster head that is closer.

The simulation results have shown the superiority of CENTRE against DSAC in terms of network lifetime, energy consumption, clustering overhead, and distance between the cluster heads and their members. These performance improvements are due to the properties of CENTRE, which are: (1) it does not require multiple beacon/data broadcasts during the cluster formation, (2) it has fixed cluster formation duration which does not depend on the number of sensor nodes, (3) it enables transmission power adjustment, (4) it has organized sleep mode, and (5) it adopts temporary support nodes which results in compact clustering. These properties make CENTRE an effective and efficient clustering method for CRSNs. In practice, we expect that CENTRE would perform the best under the condition of dense and random deployment of sensor nodes and the sensing applications that require regular data collection. However, CENTRE would need a common control channel.

## 4. Conclusions

In this paper, a novel energy-efficient and compact clustering scheme called CENTRE with temporary support nodes is proposed for CRSNs. The proposed cluster formation process has two sub-phases: cluster head discovery and cluster member invitation. Even though it is difficult for sensor nodes to find a cluster head in CRSNs, the two sub-phases enable the sensor nodes to find a cluster head efficiently. CENTRE also decreases the average distance between cluster heads and their members, resulting in compact clustering. In addition, adopting a fixed duration for cluster formation results in remarkable energy saving. The performance evaluation shows that CENTRE achieves 34% longer network lifetime with less clustering overhead. The average distance between of cluster heads and their cluster members is also decreased. The main reasons for the performance improvement of the CENTRE scheme include the following: the fixed cluster formation duration, the adjustment of the transmission power of the cluster members based on the distance to the cluster head, the refinement of the cluster formation process by the use of TS nodes, and the use of the sleep mode when the sensors are not active. In our future works, we will consider primary users involvement, backup channels selection, and network coverage analysis.

## Figures and Tables

**Table 1.** Simulation settings.

**Network Topology**	
Number of sensor nodes	120 nodes
Network area	600 m × 600 m
Sensor node's transmission range	300 m
Sensor nodes deployment	Random
Clustering setting	Probability of a sensor node becoming a cluster head (**for CENTRE**): 5%
	Optimal number of clusters (**for DSAC**): 5 clusters
**Communication Frequency**	
Frequency	Intra-cluster: IEEE 802.11 2.4 GHz ^a^
	Inter-cluster: IEEE 802.22 TV band^a^
Bandwidth	Intra-cluster: 22 MHz
	Inter-cluster: 6 MHz
Number of channels	Intra-cluster: 3 channels (any three non-overlapping channels)
	Inter-cluster: 1 channel or more (determined by the sink)
Partial spectrum sensing width (for CENTRE)	1 channel
**Timing**	
CENTRE cluster formation time	50 time slots (sub-phase 1) and 50 time slots (sub-phase 2) ^b^
CENTRE maximum delay (sub-phase 1)	25 time slots
**Power Profiles**	
Power supply	14040 J
Voltage	3 V
**Power Consumption ^c^**	
Transmit (initial)	459 μJ
Beacon	45.9 μJ
Receive	378 μJ
Active	432 μJ
Idle	172.8 μJ
Sleep	540 nJ
Sensing environment	1031.4 μJ
Partial spectrum sensing	236.9 μJ
Configuration	207.29 μJ
Spectrum switching	296.13 μJ

a Basically, cognitive radio can use the entire frequency band and this work can be extended to include a wider frequency band.

b The values are determined by considering [16].

c The values are determined by considering [17] and [18].
